# Unmelted, melted, and deconstructed Cheddar cheese: effects on the gut microbiome from a human dietary intervention study

**DOI:** 10.3389/fmicb.2026.1702111

**Published:** 2026-04-10

**Authors:** Clíona Ní Chonnacháin, Eileen R. Gibney, Emma L. Feeney, Martina Rooney, Aileen O’Connor, Nessa Noronha, Fiona Crispie, Paul D. Cotter, Jamie A. FitzGerald

**Affiliations:** 1 Food for Health Ireland, University College Dublin, Dublin, Ireland; 2 Institute of Food and Health, University College Dublin, Dublin, Ireland; 3 Teagasc Food Research Center, Moorepark, Cork, Ireland; 4 APC Microbiome Ireland SFI Research Centre, University College Cork, Cork, Ireland; 5 VistaMilk SFI Research Centre, Teagasc Moorepark, Cork, Ireland; 6 ADAPT Centre, Munster Technological University, Cork, Ireland

**Keywords:** Cheddar, cheese, diet, gut micobiome, melted, microbiome

## Abstract

**Introduction:**

Cheddar cheese is a nutritionally dense food matrix containing nutrients and bioactives with the potential to influence gut microbial characteristics. Food matrices influence nutrient absorption and digestibility, therefore the dairy matrix may affect gut microbial responses to dairy food intake. This research aims to identify gut microbial responses to Cheddar cheese consumption, considering aspects of the dairy matrix.

**Methods:**

Secondary analysis was conducted on a subset (*n* = 69) of participants’ data collected during a 6-week parallel 3-armed intervention study. Interventions involved daily consumption of one of the following: (A) 120 g unmelted Cheddar cheese; (B) 120 g melted Cheddar cheese; (C) butter (49 g), calcium caseinate powder (30 g), and Ca supplement (500 mg). Demographics, anthropometry, dietary intake and fecal samples were collected at baseline (V1) and post-intervention (V2). Fecal samples underwent 16S rRNA gene sequencing, followed by bioinformatic processing and statistical analysis.

**Results:**

At V1, 52% were female, mean age was 58.2 ± 5.4 years, with no significant differences between groups or timepoints. Following sequencing, 12,098 unique bacterial taxa in total were identified. Under a False Discovery Rate (FDR) cutoff of 0.1, *Dorea* (*W* = 0.568, FDR = 0.079) and *Erysipelotrichaceae UCG-003* (*W* = 0.887, FDR = 0.097) were significantly increased from V1 to V2 in the unmelted cheese group. At V2, *Bacteroides* was differentially more abundant in the unmelted cheese group, relative to the melted group (*W* = 0.587, FDR = 0.034). Bacterial alpha diversity (Shannon, Simpson) significantly increased in the unmelted cheese group only from V1 to V2 (*p* < 0.05). Beta diversity analysis showed a significant group effect considering both timepoints (*F* = 1.505, *p* < 0.01). Considering V2 only, Principal Coordinate Analysis showed the unmelted group clustered more closely relative to the other groups, although the effect was not significant.

**Discussion:**

Unmelted Cheddar cheese modulated the gut microbiome by increasing alpha diversity and abundance of several fermenting bacteria. Overall community structure also became more similar following consumption of unmelted cheese, relative to the other groups. Heating cheese and altering its physical structure disrupts the dairy matrix, potentially influencing downstream gut-nutrient interactions and subsequent gut microbial response.

## Introduction

Cheese is a nutrient-rich food which provides macronutrients including bioavailable protein and lipids, and micronutrients including calcium, phosphorus, vitamin A, riboflavin and vitamin B12 (Haug et al., 2007). Dietary intake data from Ireland, the UK, and the US show that cheese is an important source of these nutrients (Feeney et al., 2016). Annual global cheese production has steadily increased over recent years, further highlighting its relevance in the modern diet (Zheng et al., 2021). A wide variety of cheeses are available, produced through different processing and ripening techniques, affecting their physical, nutritional and bioactive characteristics (Zheng et al., 2021). Starting with milk, cheesemaking involves the following universal processing steps: inoculation, coagulation, curd cutting (separating curds and whey) and draining whey (Zheng et al., 2021). Depending on the cheese type (e.g., hard ripened cheese such as Cheddar, or soft fresh cheese such as ricotta) additional steps in processing can involve pasteurization, curdling, cooking, salting, pressing and ripening (Zheng et al., 2021). Inoculation is the addition of a starter culture (e.g., lactic acid bacteria), causing fermentation, where desirable microbial action can provide bioactive compounds (e.g., exopolysaccharides, peptides) and, potentially, probiotic properties (Awad et al., 2005; López-Expósito et al., 2017; Marco et al., 2021). In addition to fermentation during processing, in ripened cheeses (e.g., Cheddar, Parmesan), the ripening step can also allow microbial growth, encouraging the production of probiotic microbes such as non-starter lactic acid bacteria (NSLAB) (Leeuwendaal et al., 2021). Bioactive and other fermentation-derived dairy compounds have the potential to influence health beyond the scope of benefits from non-fermented dairy products (Leeuwendaal et al., 2022). This is particularly relevant in the context of gastrointestinal health, wherein fermented foods can positively influence the gut microbiome and reduce symptoms of dysfunction (Leeuwendaal et al., 2022).

Consumption of fermented foods in human societies dates back to ancient times, with the first evidence of food fermentation recovered from 7000 BC (McGovern et al., 2004). The importance of fermented foods in the modern diet has gained traction in recent years, with calls to include them in food-based dietary guidelines (Chilton et al., 2015). Fermented dairy products, in particular, are known to influence many aspects of the gut microbiome (Kato et al., 2004; Li et al., 2020; Liu et al., 2015, 2017; Ní Chonnacháin et al., 2024; Rabah et al., 2020; Sprong et al., 2010; Veiga et al., 2014; Yan et al., 2020; Yilmaz et al., 2019). Human intervention studies show fermented milk, yogurt, and kefir increase gut microbial diversity, and can also increase or decrease certain commensal or pathogenic bacterial strains, respectively (Kato et al., 2004; Li et al., 2020; Liu et al., 2015; Veiga et al., 2014; Yilmaz et al., 2019). Similar effects are seen in *in vitro* and animal models fed with milk powders and cheeses (Bottari et al., 2017; Kim et al., 2020; Liu et al., 2017; Rabah et al., 2020; Sprong et al., 2010; Yan et al., 2020). However, relatively few human dietary intervention studies to date have explored the effect of cheese consumption on the gut microbiome. Firmesse et al. (2007) showed that Camembert cheese consumption (40 g, twice daily) increased levels of commensal enterococci in stool following a 4-week intervention in a healthy human cohort. Milani et al. (2019) demonstrated that bacteria harbored in Parmesan cheese colonized the human gut following a 7-days consumption period (45 g/day) in a healthy cohort. Human intervention studies investigating the influence of other widely consumed ripened cheeses (e.g., Cheddar) on the gut microbiome are limited. Cheddar cheese is of particular importance due to its high consumption in Western populations, and potential to influence the gut microbiome (Department for Environment, Food & Rural Affairs, and Office for National Statistics (UK), 2022; Feeney et al., 2015; United States Department of Agriculture Economic Research Service, 2024; Zheng et al., 2021). When Cheddar cheese ripens, NSLAB grows within the cheese matrix (Leeuwendaal et al., 2021). Leeuwendaal et al. (2021) demonstrated that NSLAB which develop during the ripening of Cheddar cheese harbors potentially beneficial bacteria, which resist digestion and establish themselves in an *in vitro* gastrointestinal model. Specifically, representatives of two *Lactobacillus* species (*Lactobacillus paracasei*, *Lactobacillus rhamnosus*) survived simulated gastrointestinal transit and inhibited binding of pathogenic *Escherichia coli* (Leeuwendaal et al., 2021). This suggests that NSLAB present in Cheddar cheese can interact with the gut microbiome, potentially conferring further benefits to the host (Leeuwendaal et al., 2021).

When considering the link between dairy foods and health, it is important to consider the food matrix, which has received significant attention in nutrition research (Aguilera, 2019). The concept of the food matrix effect is that the physical structure wherein nutrients are situated can influence digestion and absorption of nutrients contained within the food, subsequently influencing downstream biological responses (Aguilera, 2019). Dairy food matrices, including cheese, are highly heterogenous in both nutritional properties and structural aspects. This can be a result of initial processing affecting nutritional and structural properties (e.g., Cheddar vs. cottage cheese), through to the mode of consumption (e.g., melted vs. unmelted Cheddar cheese), which can alter the structural matrix of the same food product (Thorning et al., 2017). Previous studies show that aspects of the dairy matrix (e.g., physical structure) can influence biological responses to dairy consumption, including differences in cardiometabolic, skeletal and body compositional responses, as well as in digestion kinetics (Aguilera, 2019; Geiker et al., 2020; Thorning et al., 2017). In addition, research in other foods such as almonds has shown that food matrix alteration through processing (e.g., whole almond vs. almond butter) influences downstream gut microbial responses (Holscher et al., 2018). It is therefore plausible that altering the dairy food matrix may also modulate gut microbial responses associated with consumption. Cheddar cheese is often consumed in a melted form (e.g., lasagne, grilled cheese sandwiches), wherein the structural matrix is altered and disrupted through heating (O’Connor et al., 2024). Considering that the Cheddar cheese structural matrix is commonly altered prior to consumption, the effect of this on gastrointestinal outcomes (e.g., gut microbiome characteristics) warrants exploration. This considered, we hypothesized that Cheddar cheese may influence gut microbial characteristics, and that this may be modulated by the food matrix effect. The aim of this work was to investigate changes in gut bacterial abundance and diversity following Cheddar cheese consumption, and to explore the effect of altering the cheese matrix prior to consumption (unmelted, melted, deconstructed state).

## Materials and Methods

### Participants

**FIGURE 1:**
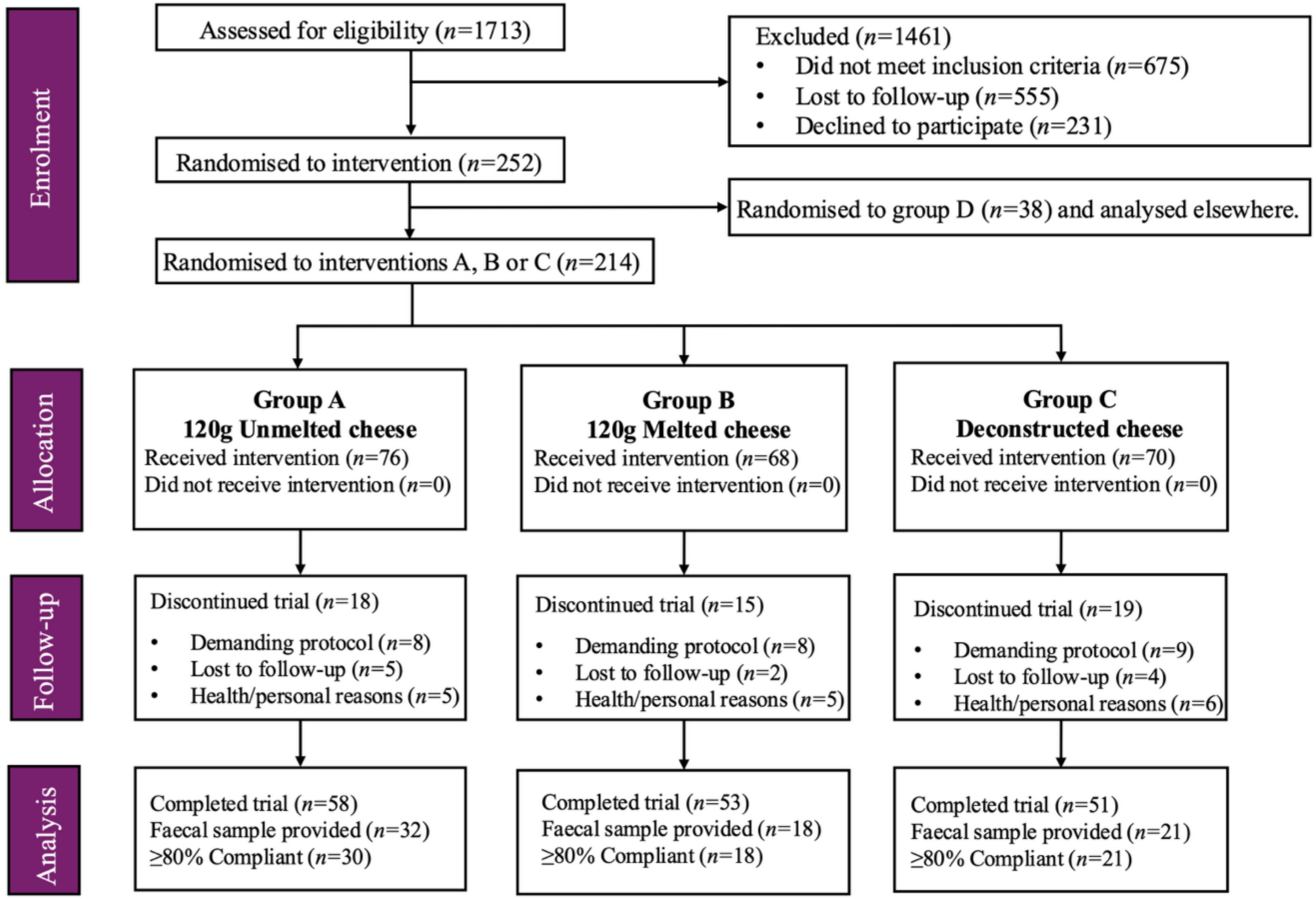
Participant flowchart. Of the *n* = 252 participants assigned to the intervention, *n* = 162 completed Group A, B and C. Of those, *n* = 71 provided an optional fecal sample for the current analysis, of which *n* = 69 were ≥80% compliant with the study protocol. Group D was part of the original study design and will be compared to group A in separate analysis, as outlined in the study design and trial registry. Adapted from O’Connor et al. (2024).

Within the work presented here, analysis was conducted on a subset (*n* = 69) of participants from a larger 6-week parallel dietary intervention study (O’Connor et al., 2024). The primary outcome of the larger study was to determine the impact of the cheese matrix structure (unmelted, melted, deconstructed state) on blood lipid concentrations, which determined the participant inclusion and exclusion criteria. Inclusion criteria were age ≥ 50 years, body mass index (BMI) ≥ 25 kg/m^2^, no chronic co-morbidities, freedom from dairy allergy/intolerance and consumption of an omnivorous diet. Participants were excluded if taking cholesterol/blood pressure medication, undergoing prescribed or therapeutic diets, or actively trying to lose weight. Participants were screened based on inclusion/exclusion criteria using an online questionnaire. All participants provided written informed consent prior to study participation. A total of 252 participants were recruited between January 2020 and December 2022 in Dublin, Ireland. This paper focuses on the cohort subset (*n* = 69) of the overall study cohort who optionally provided a fecal sample for use in this analysis (Figure 1). All study protocols were approved by the University College Dublin (UCD) Human Research Ethics Committee (LS-19-78-Gibney) and the trial was registered at ISRCTN as ISRCTN11913510.

### Study design

The overall study was a 6-week parallel 4-arm dietary intervention trial. Figure 1 outlines the overall study participant flowchart. Participants (*n* = 252) were randomized to one of four dietary interventions for a 6-week period: (A) 120 g/day of unmelted, pasture-fed, full-fat cheddar cheese; (B) 120 g/day melted, pasture-fed, full-fat cheddar cheese; (C) butter (49 g), calcium caseinate powder (30 g), and a calcium (CaCO_3_) supplement (500 mg), (D) 120 g/day unmelted, total mixed ration-fed (TMR), full-fat Cheddar cheese. Participants were randomized to dietary interventions using an online randomization tool^1^. Due to the nature of the test diets, participants and researchers were not blinded to the interventions. This current work examines groups A, B and C. Intervention Arm D (TMR) will be compared to intervention arm A elsewhere, as per the trial registry (Rooney et al., 2025). Figure 1 outlines the participant numbers of the overall study cohort (*n* = 252), where *n* = 214 were randomized to interventions A, B and C, with *n* = 162 participants completing the trial: group A (*n* = 58), B (*n* = 53) and C (*n* = 51). This cohort data was used to analyze metabolic responses to the intervention and is reported elsewhere (O’Connor et al., 2024). From groups A, B and C, a total of *n* = 71 completed the trial and provided optional fecal samples for gut microbiome analysis in this work (see Figure 1). Of these, *n* = 69 provided fecal samples (at V1 and V2), and were considered ≥80% compliant (explained below) in their respective intervention groups and thus form the subject of this paper. Supplementary Table 1 compares baseline cohort characteristics across the completers for groups A, B and C (*n* = 162), those providing a fecal sample (*n* = 71), and those providing a fecal sample which were ≥80% compliant (*n* = 69). No significant differences were identified across cohort characteristics, demonstrating the sample used for this analysis (*n* = 69) is representative of the larger study cohort.

### Intervention diets

The Cheddar cheese was provided by Tirlán (Kilkenny, Ireland). The cheesemaking process involved milk pasteurization (72°C–75°C, 16–20 s), cooling (30°C–33°C), inoculation (lactic acid bacteria), rennet addition, coagulation (30°C–33°C), heating and cooking (37°C–40°C), whey drainage and Cheddarisation, cheese cutting, curd salting, molding and packaging, storing and ripening. The cheese (groups A and B) was consumed at 8–12 months maturity for consistency across ripening stages, as this affects the cheese microbiome (Cotter and Beresford, 2017). The deconstructed cheese surrogate (group C) was specifically formulated from consumer grade butter and supplements (calcium, calcium caseinate) as a nutritionally equivalent substitute for cheese, as a negative control for the effect of the dairy matrix. Kerry Group (Cork, Ireland) provided the butter, calcium caseinate powder was purchased from Bacarel and Company Ltd., and calcium supplements were purchased from Holland and Barrett. The test diets were matched in terms of energy, protein, fat and calcium contents (Table 1). Participants in all groups were required to limit all other dairy intake to approximately 50 mL per day, but no other dietary restrictions were imposed. Participants were encouraged to incorporate the test foods into their habitual diets rather than consume them in addition to their habitual intake, to ensure they did not significantly increase their total energy intake during the intervention period. Participants in group A (unmelted) were instructed to consume the cheese in an unmelted form only. Participants in group B (melted) were instructed to consume the cheese in a melted form. Instructions for melting were to heat the 60 g cheese portions for 30 s in an 800 W microwave, or under the grill. In house testing demonstrated average temperatures more than 79°C for microwave melted cheese and 75°C for oven grilled cheese.

**TABLE 1:** Nutritional composition of the intervention diets (per day).

Group	Intervention	Energy (kcal)	Protein (g)	Fat (g)	Calcium (mg)
(A) Unmelted	120 g Irish Cheddar cheese (unmelted)	468.0	31.2	38.4	828.0
(B) Melted	120 g Irish Cheddar cheese (melted)	468.0	31.2	38.4	828.0
(C) Deconstructed	49 g butter + 30 g calcium caseinate + CaCO_3_ supplement	476.2	26.7	39.2	817.0

### Compliance

Participants received regular telephone calls to monitor compliance. Participants also filled out a daily compliance log during the intervention period to track adherence to the test diets and returned unconsumed test foods at the end of the intervention. Compliance logs were specific to each intervention arm. The cheese was packaged in daily packs containing six slices, and participants in the unmelted group logged the number of slices consumed per day. The melted group logged the method of cheese melting (e.g., microwave, grill), the number of slices melted, and reported an estimate of the amount not consumed (e.g., 1/2, 1/4) each day. For the deconstructed group, the daily test diet was one scoop (30 g) of calcium caseinate powder, eight individual portions of butter and one calcium tablet. Daily amounts of protein powder (e.g., 1/2 scoop, 1/4 scoop), number of butter portions and calcium tablets (yes/no) consumed per day were logged. Compliance over the 6-week intervention was calculated based on this data, by dividing the proportion of test food consumed by the total amount of test food per group. The analysis presented here, uses “Per Protocol” data for those who provided a fecal sample at both V1 and V2, and were deemed ≥80% compliant, in line with guidelines for nutrition intervention study reporting, and with previous studies (Feeney et al., 2018; O’Connor et al., 2024; Rooney et al., 2025; Welch et al., 2011). On this basis, two of 71 participants were excluded, providing 69 participants for this study.

### Data collection

Data was collected at baseline (V1) and after completion of the 6-week dietary intervention period (V2). Participants completed both study visits at the Human Intervention Study Suite at the Institute of Food and Health in UCD. Demographic information (age, sex) was collected. Weight and height were measured at both timepoints on a Tanita scale (Model BC-420MA) and a freestanding SECA stadiometer, respectively. Dietary intake was assessed using the EPIC-Norfolk Food Frequency Questionnaire (FFQ). Fecal sample collection kits [OMNIgene^®^GUT OM-200 (DNA Genotek Inc., Canada)] along with instructions for collection and cooler bags were posted to participants prior to their first visit. Participants were instructed to collect a fecal sample within 24 h prior to their study visit and to keep it chilled in the refrigerator until their study visit, transporting it in a cooler bag. Participants followed the same protocol for their post-intervention fecal sample. Fecal samples were then frozen at −80°C until further analysis.

### Statistical analysis (dietary intake, demographics, anthropometry)

For total energy intake, macronutrient intakes as a proportion of total energy intake, demographics, and BMI scores, data was demonstrably non-normal (skewness and kurtosis: |z| ≤ 2.58; Shapiro-Wilk *p*-values > 0.05). As a result, differences between groups at V1 for total energy intake, macronutrient intakes as a proportion of total energy intake, demographics, and BMI scores were determined by Kruskal-Wallis tests. To assess changes in these parameters over time (V1 to V2), the change (delta) in variables from visit one to visit two (V2 – V1) was calculated and compared by Kruskal-Wallis tests. Chi-square tests were used for categorical data (sex). Statistical analysis was performed using *R* (v4.4.1) using the packages dplyr and tidyr.

### Fecal microbiome analysis

Fecal samples were analyzed to determine bacterial communities through 16S rRNA gene high-throughput sequencing at the Teagasc Sequencing Center (Fermoy, Cork, Ireland). Samples were extracted using the QiaAmp Power Fecal Pro Kit (Qiagen) using manufacturer’s instructions. Samples were quantified using a Qubit Broad Range DNA kit (Thermo Fisher) and diluted to 5 ng/μl. Amplicon libraries were prepared using a miniaturization protocol based on the amplicon library preparation guide (Illumina). In brief, an Echo 525 (Beckman Coulter) was used to combine 0.5 μl DNA, 2.5 μl Kapa Hifi HotStart Ready Mix, and 1 μl of each of the primers (at 1 μM) in individual wells of a 96-well plate. The plate was spun briefly, and amplification was performed according to the amplicon library preparation guide (Illumina) with 30 cycles in place of 25. Following amplification, the volume of each sample was increased to 20 μl using molecular-grade water and the samples were cleaned using a 0.8 × ratio of Ampure beads (Beckman Coulter) on a Beckman i7 (Beckman Coulter). The index PCR was again set up using the Echo 525 to dispense the reagents as follows: 1 μl cleaned PCR product, 2 μl Unique Dual Indices (IDT), 5 μl Kapa Hifi HotStart Ready Mix, 2 μl molecular grade water. Plates were spun briefly, and PCR was performed as described in the amplicon library preparation guide (Illumina). Samples were then quantified using a Qubit High Sensitivity kit (Thermo Fisher) and pooled at equal concentration. The final pool was further cleaned using a 0.7 × ratio of Ampure beads (Beckman Coulter) and quantified using the Qubit High Sensitivity kit (Thermo Fisher). It was then sequenced on an Illumina NextSeq 2000 using NextSeq™1000/2000 P1 Reagents (600 cycles) according to the manufacturer’s guidelines (Illumina).

### Bioinformatic and statistical analysis (microbiome)

The raw sequences were downloaded from BaseSpace (Illumina) and analyzed in *R* (v4.4.1). The quality of the data was first checked using *FastQC*, and *MultiQC* was used to aggregate the reports (Andrews, 2010; Ewels et al., 2016). *Trimmomatic* (v0.39) was used to remove adapter sequences by applying ILLUMINACLIP:2:30:10:5 (Bolger et al., 2014). *FastQC* and *MultiQC* were re-run to ensure adapter removal. The DADA2 (v3.19) pipeline was then followed to produce an amplicon sequence variant (ASV) table (Callahan et al., 2016). Briefly, reads were visually quality checked, then filtered and trimmed: forward reads were truncated to 266 base pairs (bp) and reverse reads to 243 bp. To remove primers and low-quality bases, 19 bases were trimmed from the start of forward reads and 22 bases from reverse reads. The error rate model was applied followed by the core sample inference algorithm. Paired reads were then merged, the sequence table was constructed, and chimeric sequences were removed. The final ASV table was constructed by aligning sequences to the SILVA nr99 v138.1 species training set (McLaren and Callahan, 2021). The tables produced by DADA2 were then used to characterize the microbial communities of the samples in terms of bacterial abundance, alpha diversity, and beta diversity. The following packages in *R* were used: *phyloseq*, *ggplot2*, *ALDEx2*, *vegan* (Fernandes et al., 2013; McMurdie and Holmes, 2013; Oksanen et al., 2019; Wickham, 2016). Relative bacterial abundance at the genus level was calculated (raw abundances of each taxon divided by the total number of counts per sample) and visualized. As microbiome data is often sparse and non-parametric, statistical methods robust to distributional assumptions were chosen. Differences in individual taxa across intervention groups and timepoints were tested using *ALDEx2* (ANOVA-like differential expression tool for compositional data) based on the Wilcoxon rank-sum test. Differences in interaction between taxa and confounding factors (total energy and protein intakes, respectively) were checked for using the general linear model (GLM) framework for ALDEx2, according to the formulae “feature ∼ (timepoint)*(energy)*(protein)” for within-group comparisons, and “feature ∼ (Group)*(Δenergy)*(Δprotein)” for between group comparisons at V2. A thousand Monte-Carlo instances were drawn from the Dirichlet distribution for Wilcoxon and GLM testing, and energy and protein covariates were scaled and centered. A Benjamini-Hochberg correction was applied, to control for false discovery rate (FDR). *ALDEx2* with a Benjamini-Hochberg FDR correction is conservative and has high precision, although has lower sensitivity and a risk of false negatives (Nearing et al., 2022), especially where effects are subtle or sample size is relatively low. This considered, to allow better detection of subtle changes in differentially abundant taxa, FDR < 0.1 was considered statistically significant. For *ALDEx2* testing, a filtered subset was used for the analysis by removing taxa not seen at more than 0.5% abundance in at least 10% of samples. Species richness was calculated by determining the number of distinct ASVs detected per sample and were compared across all three groups using a Kruskal-Wallis test. Alpha diversity was calculated as per Shannon and Simpson indices. Differences over time within groups (V1 vs. V2) were compared using Wilcoxon signed-rank tests. Delta variables (change in Shannon/Simpson index over time) were used to compare changes in alpha diversity between the intervention groups (V2 vs. V2), in a pairwise manner, using Wilcoxon rank-sum tests. Beta diversity was analyzed by calculating a Bray-Curtis (BC) dissimilarity matrix, and plotted by Principal Coordinate Analysis (PCoA) based on two Principal Components (PC): PC1 and PC2. Permutational multivariate analysis of variance (PERMANOVA) was performed on the BC matrix with 999 permutations, to assess differences in beta diversity across groups and timepoints, accounting for the scaled, centered effects of energy and protein intake; age and sex were considered as covariates throughout the analysis. The sequence data is available on the European Nucleotide Archive under project accession number PRJEB89773.

## Results

### Cohort descriptives and dietary intake

A total of 71 participants provided fecal samples at V1 and V2 for this work. This included *n* = 32 in the unmelted cheese group, *n* = 18 in the melted cheese group and *n* = 21 in the deconstructed cheese group. Of these, participants who were ≥80% compliant were included in the fecal microbiome analysis (*n* = 69). This included *n* = 30 in the unmelted cheese group, *n* = 18 in the melted cheese group and *n* = 21 in the deconstructed cheese group. The average total compliance for the groups in the analysis presented here was 98%, with 94% compliance in the unmelted group, and 100% in the melted and deconstructed groups. Of the cohort presented here (*n* = 69), 52% were female, mean age was 58.3 years, and mean BMI was 28.3 kg/m^2^ at baseline (Table 2). At baseline, there were no significant differences terms of sex distribution, age or BMI across intervention groups. Mean energy intakes were 2245.72 and 1907.30 kcal at baseline and post-intervention, respectively. No differences in either total energy or macronutrient intakes (proportionate to total energy intake) were observed at baseline across intervention group. Protein intake (% total energy intake) for the deconstructed group significantly decreased from V1 to V2 with respect to the changes seen in the unmelted and melted groups (*p* < 0.001), both of which demonstrated a slight increase (Table 2). Energy intake was notably higher in the post-intervention deconstructed group, although the difference was not significant.

**TABLE 2:** Cohort descriptives and dietary intake (*n* = 69).

	Unmelted (*n* = 30)		Melted (*n* = 18)		Deconstructed (*n* = 21)		*p* ^a^	*p* ^b^
Sex	*n*	%	*n*	%	*n*	%		
Male	13	41.94	11	64.71	9	42.86	0.273	–
Female	18	58.06	6	35.29	12	57.14		
	**Mean**	**SD**	**Mean**	**SD**	**Mean**	**SD**		
Age (years)	57.78	6.06	58.94	5.29	58.38	4.70	0.663	–
BMI (kg/m2)								
Baseline	28.66	3.56	28.05	3.34	28.11	3.23	0.882	0.063
Post	28.56	3.59	27.79	3.25	28.16	3.22		
Energy (kcal)								
Baseline	2261.14	940.35	2180.78	974.84	2277.02	1008.36	0.828	0.121
Post	1755.17	557.99	1908.66	805.92	2345.41	1626.31		
CHO (% TE)								
Baseline	44.18	4.56	44.60	5.97	40.92	7.64	0.350	0.090
Post	38.52	12.00	41.81	8.81	42.54	8.92		
Protein (% TE)								
Baseline	16.86	3.11	17.57	3.63	17.14	2.47	0.831	<0.001*
Post	19.41	2.68	20.26	5.04	14.32	2.78		
Fat (% TE)								
Baseline	38.32	5.89	38.23	4.17	31.37	5.47	0.131	0.062
Post	41.66	8.61	38.57	6.37	43.71	7.87		

*p*^a^ indicates differences between groups at baseline (Kruskal-Wallis or chi-square tests, where appropriate).

*p*^b^ indicates differences over time between groups (Kruskal-Wallis test).

*Significant difference in protein intake between groups A and C (*p* < 0.001) and B and C (*p* < 0.001). SD, standard deviation; BMI, body mass index; CHO, carbohydrate; % TE, proportion of total energy intake (%).

### Community composition and abundance

**FIGURE 2:**
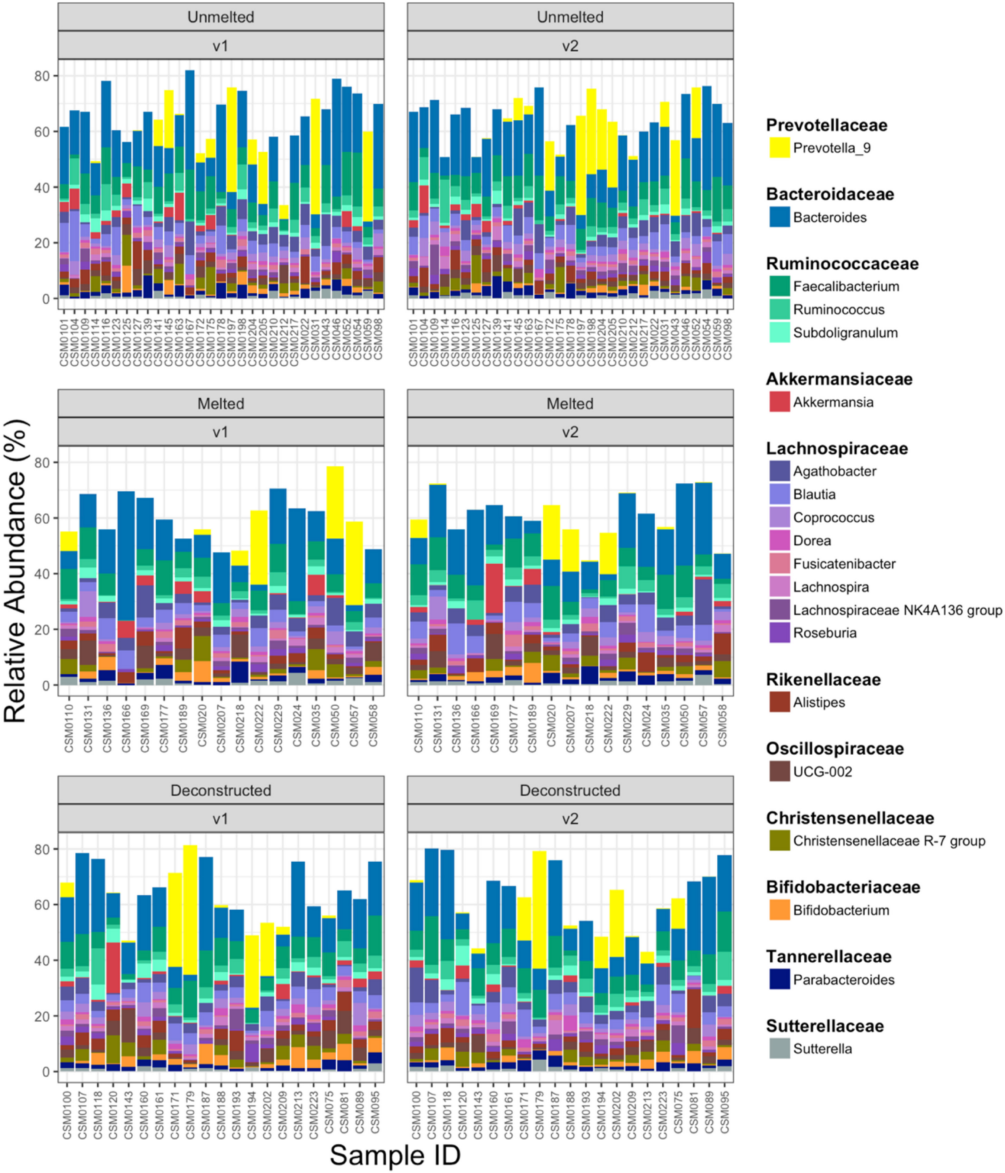
Relative bacterial abundance. The 20 most abundant bacterial genera grouped and colored by family in each intervention group, split by timepoint in *n* = 69 participants. Empty space represents genera outside of the 20 most abundant genera. v1, baseline; v2, post-intervention.

Across the total cohort and considering both timepoints, 12,098 unique bacterial ASVs were identified. Figure 2 illustrates the 20 most abundant genera identified in each intervention group at an individual sample level, split by timepoint. Samples are ordered based on Bray-Curtis dissimilarity. Taxonomic composition was consistent with that of the human gut microbiome, with dominant genera including *Bacteroides* (17.14% total abundance), *Faecalibacterium* (8.47% total abundance) and *Prevotella_9* (6.27% total abundance) (Greenhalgh et al., 2016). Under an FDR (Benjamini-Hochberg) cutoff of 0.1 (see Section “Materials and methods”), the relative abundance of *Dorea* (*W* = 0.568, FDR = 0.079) and *Erysipelotrichaceae UCG-003* (*W* = 0.887, FDR = 0.097) significantly increased in the unmelted cheese group from V1 to V2. *Bacteroides* was differentially more abundant in the unmelted group, relative to the melted cheese group, at V2 (*W* = 0.587, FDR = 0.034). A single *Lachnoclostridium* taxon increased significantly with protein and energy intake (Table 2) in the deconstructed group at V2 (FDR 0.02 – 0.09). No other taxa were differentially abundant over time within the melted or deconstructed groups, or between the intervention groups, following FDR correction.

### Alpha diversity

**FIGURE 3:**
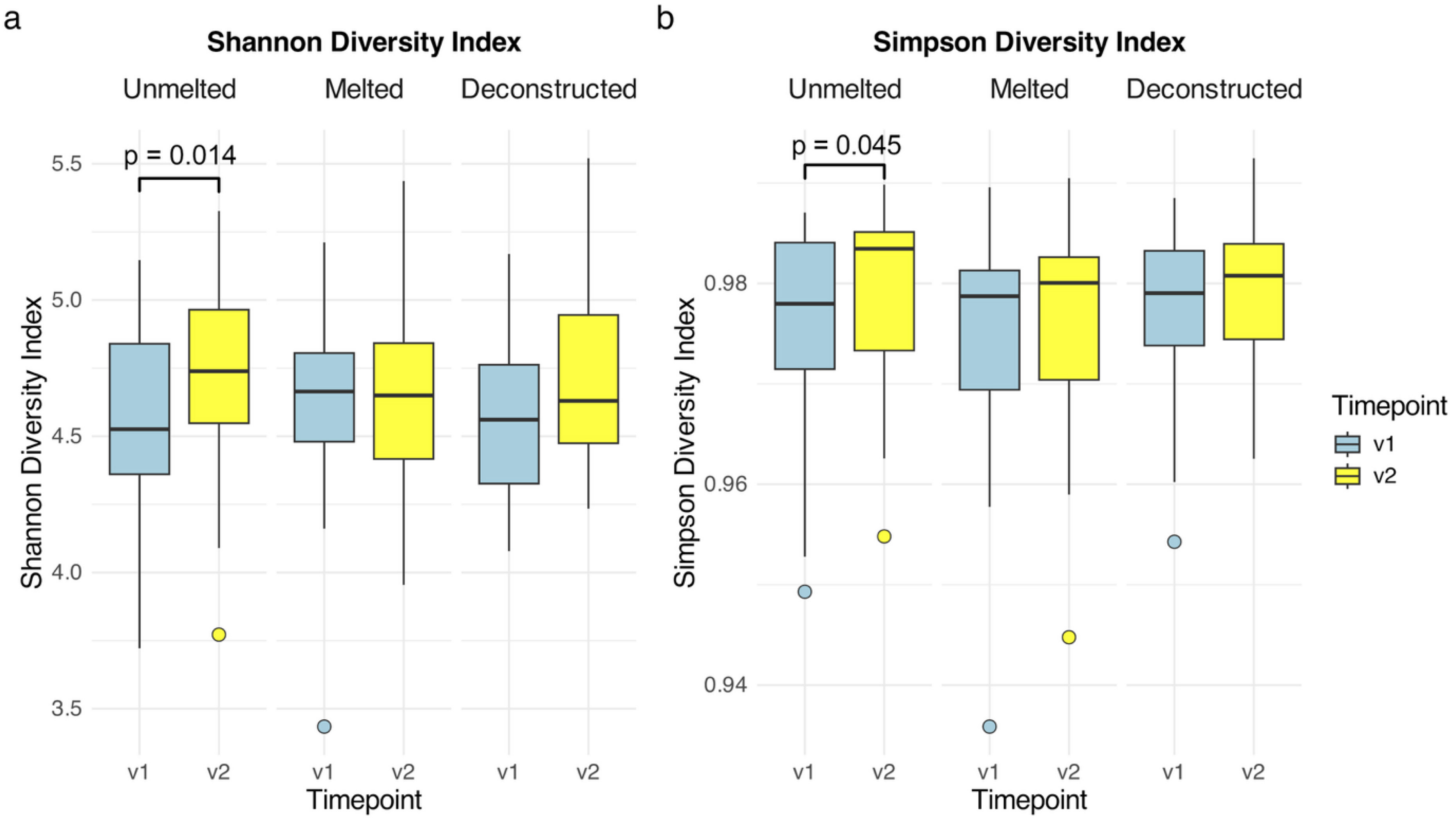
Alpha diversity by group and timepoint. Shannon and Simpson alpha diversity split by group and timepoint in *n* = 69 participants. **(a)** Shannon diversity by group and timepoint; **(b)** Simpson diversity by group and timepoint. v1, baseline; v2, post-intervention.

Bacterial richness based on observed ASVs per sample ranged from 224 to 1009, with a mean of 557.06 ± 158.33. There were no differences in the observed ASVs detected between the intervention groups overall at both timepoints, at V1 or at V2 (*p* > 0.300). Significant changes in alpha diversity were observed within the unmelted group only, where both Shannon and Simpson diversity increased from V1 to V2 (Figure 3: Shannon: *V* = 124, *p* = 0.014; Simpson: *V* = 146, *p* = 0.045). There were no differences in alpha diversity over time within the melted or deconstructed groups, or between the intervention groups.

### Beta diversity

**FIGURE 4:**
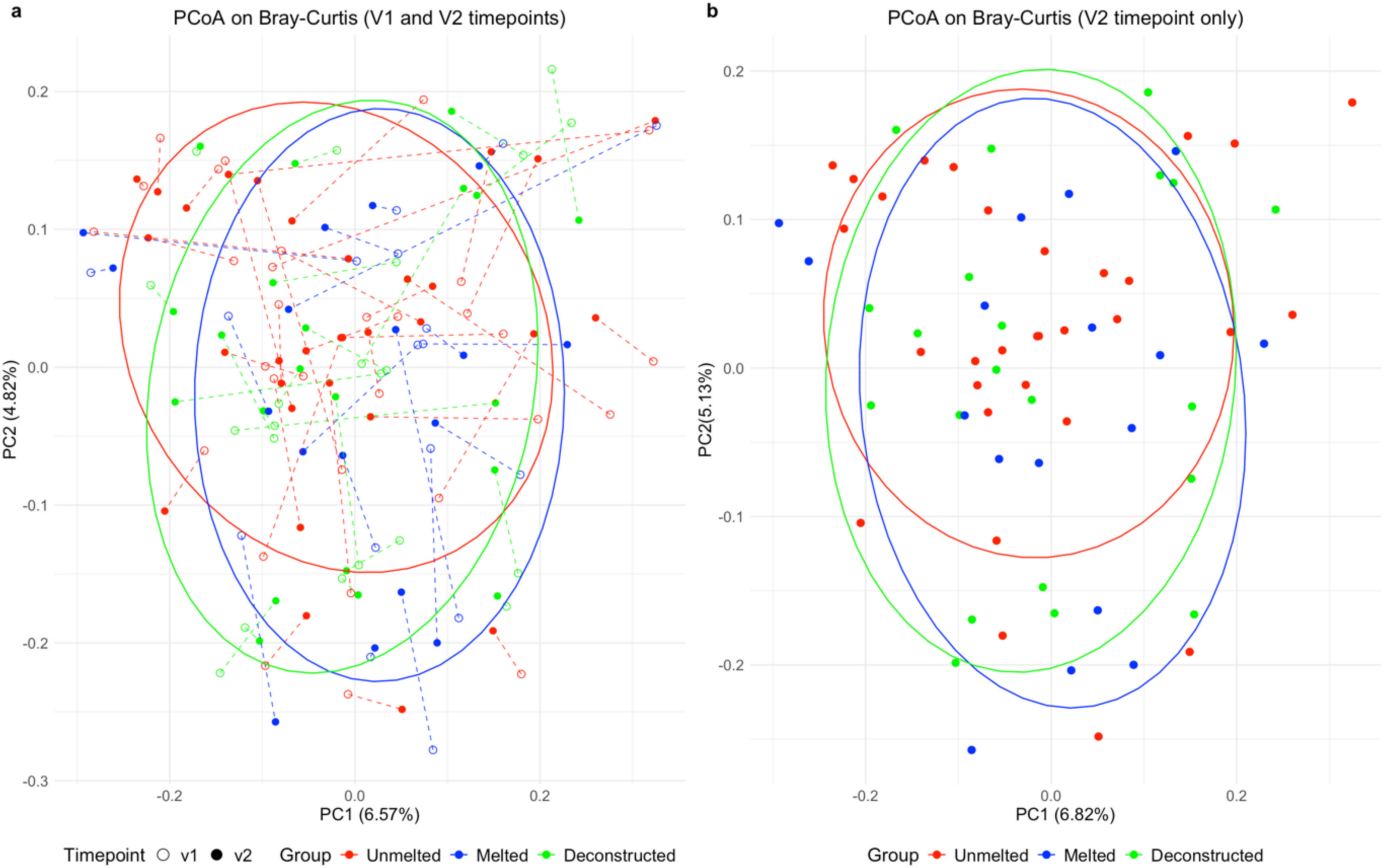
Beta diversity plots. Beta diversity by Principal Coordinate Analysis on Bray-Curtis in *n* = 69 participants: **(a)** Principal Coordinate Analysis on Bray-Curtis dissimilarity at both timepoints; **(b)** Principal Coordinate Analysis on Bray-Curtis dissimilarity at V2 only. Ellipses represent 80% confidence intervals. PCoA, Principal Coordinate Analysis; PC1, principal component 1 axis; PC2, principal component 2 axis; v1, Baseline; v2 post-intervention.

Beta diversity was used to evaluate the effect of the intervention on microbiome community structure, with Figure 4a illustrating the two largest components of microbiome variation as the X (principal component 1, 6.5%) and Y (principal component 2, 4.8%) axes, respectively, considering both V1 and V2. The food matrix effect can be seen in the PCoA plot for V2 (Figure 4b), reflecting response to the intervention, where unmelted samples cluster more closely together (red) while both melted and deconstructed samples are spread over a wider area (blue and green, respectively), showing a higher degree of variability in the structure of those participants’ microbiomes after exposure to those test foods. When tested (using PERMANOVA of group, timepoint, energy and protein intake), the microbiomes of different intervention groups are significantly distinct from one another (*F* = 1.49, *p* = 0.001), with the group and timepoint factors explaining 2.8% of variability seen in microbiome structure across this study. Sub-setting the data to focus on the explicit effect of treatment only (by excluding treatment-naïve V1 samples), the experimental grouping (unmelted, melted, deconstructed) accounted for 3.1% of the difference in microbiomes at V2 (6.82% and 5.13% of variation for principal components 1 and 2, respectively). However, this subset showed no significant differences between the groups when tested (PERMANOVA: *F* = 1.034, *p* = 0.364), and subsetting by intervention group showed no within-group differences over time (V1 – V2 by group; PERMANOVA, *p* > 0.15).

## Discussion

This intervention study examined the influence of Cheddar cheese consumption on gut microbiome composition, while also investigating the effects of altering the food matrix. Our analysis demonstrated that consumption of unmelted Cheddar cheese significantly increased bacterial alpha diversity, with increases seen in the fermenting bacteria *Dorea* and uncharacterized *UCG-003 Erysipelotrichaceae*. This suggests unmelted Cheddar cheese may positively modulate the gut microbiome by increasing species richness and evenness, and the abundance of specific taxa (Leeuwendaal et al., 2022). Additionally, *Dorea* and *Bacteroides* have established roles as fermenting microbes, with previous studies demonstrating their capability to ferment dietary substrates (e.g., fiber) and produce beneficial metabolites, notably short-chain fatty acids (SCFAs) such as butyrate and acetate (Taras et al., 2002; Wexler, 2007). These SCFAs are considered beneficial in the colonic environment and are associated with gut and immune homeostatic maintenance (Tan et al., 2014). While the metabolic activity of the genus *Erysipelotrichaceae UCG-003* has not been thoroughly characterized to date, at the family level, *Erysipelotrichaceae* are shown to be fermenters and producers of beneficial SCFAs, including butyrate and acetate (Tegtmeier et al., 2016). Therefore, considering the individual taxa which increased in response to unmelted Cheddar cheese consumption, these microbes have the potential to benefit the GI environment due to their capacity to ferment dietary substrates and produce beneficial metabolites (Tan et al., 2014). Consumption of unmelted Cheddar cheese also increased bacterial alpha diversity, which is an important finding as a loss of gut bacterial alpha diversity is associated with a host of conditions including immune, metabolic, and digestive disorders (Mosca et al., 2016). Identifying foods or diets which can promote a diverse gut microbiome could ameliorate symptoms related to these conditions, underscoring the importance of characterizing this effect for unmelted Cheddar cheese.

Interestingly, an increase in bacterial alpha diversity was not demonstrated when the same Cheddar cheese was consumed in a melted form, while *Bacteroides* was also significantly more abundant in the unmelted group, relative to the melted group after the intervention. One explanation for these differences is that the structural delivery matrix of the cheese encapsulating the nutrients, potential probiotics and bioactives may influence digestion, downstream gut-nutrient interactions, and subsequent gut microbial response. In terms of nutrient and probiotic delivery, the cheese matrix has demonstrated several beneficial attributes by creating a buffering effect against the highly acidic gastrointestinal environment and by providing a structurally dense, protein- and fat-rich matrix acting as a protective barrier (Gomes da Cruz et al., 2009; Leeuwendaal et al., 2021; Sharp et al., 2008). When Cheddar cheese is melted, the dense semi-solid structural matrix is disrupted and becomes a looser viscoelastic food structure (Thorning et al., 2017). Considering the role of the dense structural matrix which protects the nutrients and bioactives encapsulated in the cheese, this may partly explain why we don’t see an increase in alpha diversity or bacterial taxa in the melted cheese group. In addition, participants in the melted group heated the cheese to approx. 75°C–79°C, which may have also degraded beneficial microbes (e.g., NSLAB with probiotic potential) present in the cheese (Leeuwendaal et al., 2021). Although thermotolerance varies between different microbial species, heat stress above 50°C is generally harmful to microbes present in foods (Tripathi and Giri, 2014). Thus, by melting the cheese, the dual effect of disrupting the structural matrix and causing heat stress to potentially beneficial microbes may explain the differential response in the cheese groups, in terms of bacterial alpha diversity and bacterial abundance. However, these effects warrant further exploration.

A large body of research shows that processing and altering the bovine milk matrix profoundly influences downstream biological responses and health outcomes (Thorning et al., 2017). One of the current research gaps in this area, identified by Weaver, poses the question: “*What is the impact of the dairy matrix on gut microbiome composition and function?*” (Weaver, 2021). In this study, the deconstructed cheese group is nutritionally equivalent to the cheese groups, although it is not cheese. It is significantly different to Cheddar cheese in terms of processing steps (notably fermentation) and structural aspects, so serves as a nutritionally equivalent comparator in this study. With the exception of a single fermenting taxon (*Lachnoclostridium*) which increased alongside elevated energy and protein intake, the deconstructed group did not significantly alter gut microbial characteristics (alpha diversity, relative bacterial abundance), unlike the unmelted cheese group. The effect of the deconstructed cheese group on the gut microbiome was more in line with the melted cheese group, which also saw no significant change. As noted, in the melted group, the melting process disrupts the physical matrix, and also likely disrupts the potentially beneficial microbes and bioactives produced during cheese-making (notably fermentation and ripening steps). This considered, the findings here suggest that properties of the cheese matrix (fermentation, physical structure) influence gut microbial responses associated with consumption. This supports the current thinking that fermentation and physical structure are important properties of the dairy matrix influencing biological responses to dairy food consumption (Geiker et al., 2020; Thorning et al., 2017; Weaver, 2021). In line with previous work, this also reinforces the importance of studying different food matrices and forms of the same food, particularly in which they are consumed, alongside considering the differing nutritional compositions of such foods (Weaver, 2021). Elsewhere, the importance of the dairy matrix effect is well-established across multiple biological parameters (e.g., cardiovascular, skeletal), and based on this research, it may prove an important consideration for future gut microbiome studies (Geiker et al., 2020).

Unmelted Cheddar cheese consumption led to increased microbial (alpha) diversity, with a significant difference in overall microbiome composition between groups (beta diversity) only when considering all samples (including treatment-naïve V1 samples). PCoA showed a visual distinction between groups, where at V2, the unmelted group clustered together more closely than the melted and deconstructed groups. This suggests that the structure of the microbiome is more similar following consumption of unmelted cheese, relative to the melted and deconstructed groups, which appear more spread across PC axes, indicating these microbiomes were more variable in their composition. However, no significant difference in microbiome composition was found between intervention groups when considering the V2 timepoint only (comparison of intervention responses). It is possible that the unmelted cheese significantly increased diversity at an individual level (alpha diversity), but the intervention did not sufficiently alter the overall community structure between groups (beta diversity) to meet statistical significance. It is also possible that the difference was not statistically significant due to the small sample size and heterogeneity between individuals (Johnson et al., 2020). Individualized responses of gut microbiota to dietary interventions are well-documented, which can complicate the identification of consistent intervention group-level gut microbial responses to dietary interventions, as captured by beta diversity (Healey et al., 2017).

There are several strengths to this intervention study, primarily due to the study design and novel research questions explored. To our knowledge, this is the first study to investigate the effect of Cheddar cheese consumption on the human gut microbiome. This is important considering dietary intake data shows Cheddar cheese is ubiquitously consumed in Western populations (Department for Environment, Food & Rural Affairs, and Office for National Statistics (UK), 2022; Feeney et al., 2015; United States Department of Agriculture Economic Research Service, 2024; Zheng et al., 2021). The importance of fermented foods in the diet is becoming increasingly well-recognized, in terms of their potential to increase both gut microbial diversity and the abundances of certain beneficial taxa (Leeuwendaal et al., 2022). Considering its palatability and wide acceptance by consumers, unmelted Cheddar cheese could be an effective vehicle for increasing fermented food intake, and increasing gut microbial diversity, especially in Western populations where other fermented food consumption (e.g., kefir, sauerkraut) can be low (Chilton et al., 2015). Furthermore, this is the first study to consider the dairy matrix effect in the context of gut microbial response to cheese. As cheese is commonly consumed in a melted form, here we address a practical research question in the context of realistic dietary intake, in an effort to maximize the potential for Cheddar cheese to support the microbiome (O’Connor et al., 2024). It is critical that we understand how the melting of cheese may negatively impact the gut microbiota-altering potential of Cheddar cheese. In the interest of promoting gut health through dietary intake, this knowledge may influence consumers to consume Cheddar cheese in an unmelted state to protect the food matrix prior to digestion. Although further studies employing larger sample size would be valuable in clarifying the role of the cheese matrix, this human dietary intervention indicates that consumption of unmelted Cheddar cheese positively influences the composition of the gut microbiome.

Due to the secondary nature of this study, there are several important limitations to consider. Sample size is a limitation, and this work relied on samples provided on an optional basis within a larger study. As a result, an *a priori* power calculation for the primary outcome of the work presented here (changes to gut microbial structure) was not performed. However, this exploratory analysis now provides data and information that may be used to power future studies. The study cohort was also determined based on the primary outcome of the larger study primarily investigating blood lipid response, leading to a reduced sample size and uneven split between intervention groups (O’Connor et al., 2024). The subjects within this study were overweight and ≥50 years old, which may limit application of these findings to a wider population (Le Chatelier et al., 2013). Dietary intake data was collected by FFQ, which provided estimated total energy and macronutrient intakes. While FFQs are convenient in terms of participant and researcher burden, they provide limited detail to control for background dietary intake in this type of study (Johnson et al., 2020). Although no differences in dietary intake were observed over time within or between intervention groups based on the FFQ data, it is possible that dietary factors beyond the scope of the FFQ influenced gut microbial outcomes. Similarly, while great care was taken to match the composition of cheese, there may be unidentified factors in the deconstructed cheese which differentiate its dietary impact from that of its melted or solid state. Lastly, our choice of FDR threshold (FDR < 0.1) for differentially abundant taxa may also be considered a limitation due to being slightly more lenient and should be interpreted accordingly. As mentioned, *ALDEx2* with a Benjamini-Hochberg correction is stringent and is associated with type 2 errors, which can make it difficult to detect subtle changes in the gut microbiome, particularly in small sample sizes (Nearing et al., 2022). Nonetheless, this study has uncovered interesting and novel findings and provides a rationale to further explore the central research question as a primary outcome in larger cohorts.

This study demonstrates the potential for unmelted full-fat Cheddar cheese to interact with the gut microbial environment, increasing bacterial alpha diversity and the abundance of several fermenting microbes. In contrast, Cheddar cheese in a melted form, and its nutritional components in a deconstructed form, affected neither alpha diversity nor bacterial abundance. Additionally, the dietary intervention differentiated microbial community structure between groups, wherein the unmelted group clustered more closely in response to the intervention, relative to the melted and deconstructed groups. This research shows that the gut microbiome responsive to alterations in dairy food matrices, specifically with respect to fermentation and structural properties. In line with previous studies, altering the dairy food matrix influences the downstream biological response (Thorning et al., 2017; Unger et al., 2023; Weaver, 2021). For Cheddar cheese, a clearer understanding of the interaction between intake and the gut microbiome which considers the food matrix will be advantageous and insightful for public health initiatives (e.g., including fermented foods in dietary intake guidelines), and for optimizing gut health through dietary strategies.

## Data Availability

The datasets presented in this study can be found in online repositories. The names of the repository/repositories and accession number(s) can be found below: https://www.ebi.ac.uk/ena, PRJEB89773.

## Footnotes

1
https://www.sealedenvelope.com
