# Supplementary material for: Unmelted, melted, and deconstructed Cheddar cheese: effects on the gut microbiome from a human dietary intervention study
Source: Front Microbiol. 2026 Apr 10;17:1702111. doi: 10.3389/fmicb.2026.1702111 (PMC13106160; doi:10.3389/fmicb.2026.1702111)
Supplement: Supplementary file 1 [file Table_1.docx]

## Supplementary information

**Supplementary Table 1.** Baseline characteristics across cohorts.

|  | **Total completers** (*n=*162) | | **Faecal samples provided** (*n=*71) | | **≥80% compliant**  (*n=*69) | | **p** |
| --- | --- | --- | --- | --- | --- | --- | --- |
| **Sex** | ***n*** | **%** | ***n*** | ***%*** | ***n*** | **%** |  |
| Male | 70 | 43.21 | 34 | 47.89 | 33 | 47.83 | 0.721 |
| Female | 92 | 56.79 | 37 | 52.11 | 36 | 52.17 |  |
|  | **Mean** | **SD** | **Mean** | **SD** | **Mean** | **SD** |  |
| **Age (years)** | 58.40 | 5.78 | 58.25 | 5.47 | 58.25 | 5.43 | 0.983 |
| **BMI** | 28.74 | 4.36 | 28.41 | 3.36 | 28.34 | 3.37 | 0.982 |
| **Energy (kcal)** | 2206.71 | 1153.96 | 2388.09 | 1550.79 | 2245.72 | 955.35 | 0.823 |
| **CHO (%TE)** | 42.82 | 6.66 | 43.37 | 6.00 | 43.33 | 6.07 | 0.787 |
| **Protein (%TE)** | 17.17 | 3.04 | 17.07 | 3.04 | 17.12 | 3.05 | 0.991 |
| **Fat (%TE)** | 39.67 | 5.89 | 39.23 | 5.51 | 39.19 | 5.50 | 0.924 |

p indicates differences across groups at baseline (Kruskal-Wallis or chi-square tests, where appropriate). SD, standard deviation; BMI, body mass index; CHO, carbohydrate; %TE percentage of total energy intake. Total completers (*n*=162) denotes participants who completed arms A, B and C of the overall intervention study which provided data for this work. Faecal samples provided (*n*=71) denotes participants within that study cohort who provided a baseline and post-intervention faecal sample. ≥80% Compliant (*n*=69) denotes participants who provided a baseline and post-intervention faecal sample and adhered to the intervention at a compliance rate ≥80%.
